# Altruistic behavior in cohesive social groups: The role of target identifiability

**DOI:** 10.1371/journal.pone.0187903

**Published:** 2017-11-21

**Authors:** Ilana Ritov, Tehila Kogut

**Affiliations:** 1 School of Education and Center for the Study of Rationality, Hebrew University, Jerusalem, Israel; 2 Department of Education, and the Decision Making and Economic Psychology Center, Ben-Gurion University, Beersheba, Israel; Middlesex University, UNITED KINGDOM

## Abstract

People’s tendency to be more generous toward identifiable victims than toward unidentifiable or statistical victims is known as the *Identifiable Victim Effect*. Recent research has called the generality of this effect into question, showing that in cross-national contexts, identifiability mostly affects willingness to help victims of one’s own “in-group.” Furthermore, in inter-group conflict situations, identifiability increased generosity toward a member of the adversary group, but decreased generosity toward a member of one’s own group. In the present research we examine the role of group-cohesiveness as an underlying factor accounting for these divergent findings. In particular, we examined novel groups generated in the lab, using the minimal group paradigm, as well as natural groups of students in regular exercise sections. Allocation decisions in dictator games revealed that a group’s cohesiveness affects generosity toward in-group and out-group recipients differently, depending on their identifiability. In particular, in cohesive groups the identification of an in-group recipient decreased, rather than increased generosity.

## Introduction

Contrary to the strict rational agent model, people are not solely concerned with maximizing their own benefits in resource allocation situations. Often, as has been documented in a large volume of research [1; 2; 3; 4], people engage in altruistic giving to strangers. Willingness to help victims who are unrelated to oneself, in situations where reciprocity is irrelevant, is a common form of altruism. Willingness to help in these situations may be driven by self-focused attitudes such as *magical thinking* [5; 6; 7] or general moral beliefs [8; 9]. However, generosity is also affected by factors related to the recipient and to the situation in question—including the perceived neediness of the prospective recipient [4]; the perceived urgency of the need [10]; the subjective responsibility of the help-giver [11]; the singularity of the victim [12; 13; 14]; and the identifiability of the prospective recipient.

Generally speaking, acts of altruistic helping are more likely when the victim is identified [15]. Carefully controlled studies show that people are more generous toward identifiable victims than toward unidentifiable or statistical ones—even when that identification conveys no relevant individuating information [3; 11; 16: 12; 13; 17; 18; 19]. For example, Small and Loewenstein [18] found that participants playing the allocator in a dictator game sent more money to their randomly assigned recipient who had lost her endowment when she was identified by a number, than when she had not yet been determined.

Many of these studies did not explicitly place the agent or the needy victims within a specific social context. However, extensive research suggests that the group that a given victim belongs to, and the extent to which he or she identifies with that group [20; 21; 22], are important determinants of prospective donors’ willingness to help. The classification of a victim as belonging to one’s own social group arouses feelings of greater closeness and responsibility, and heightens one’s emotional response to their plight [23; 24; 25]. Willingness to help is similarly affected by social categorization: people tend to help those whom they perceive to be similar to themselves [1; 24; 26; 27; 28]. Other forms of prosociality are also affected by group categorization, and these may further interact with individual characteristics of the agents [29].

With regard to how willingness to help is affected when both social categorization and identifiability come into play, our earlier research has found that identifiability affects donor generosity in different ways, depending on the social categories evoked [30; 6]. Upon examining social categorization of victims and donors based on nationality, we found that identifiability increased helping in-group, but not out-group ones. Thus, for example, contributions toward rescuing tsunami victims were most generous when the targeted victim was described as a single compatriot who is identified by name. Similarly, expressed willingness to contribute to saving a sick child was enhanced by displaying the (same) picture of the child only when he or she was thought to be of the same nationality as the respondents. Finally, the emotions evoked by considering the victims’ plight were particularly intense when the victim was a single identified child, especially of the same nationality as the respondent.

In a more recent study [6] we examined the effect of identifiability in two other types of social categorizations: political affiliation (a group categorization relevant to inter-group conflicts) as a “hawk” (right-wing) or “dove” (left-wing) in Israeli society, and sports team supporters. In contrast with our earlier findings, in both these contexts identifiability of the recipient increased generosity toward a member of the *opponent’s* group, and decreased generosity toward a member of one’s own group.

The apparent reversal of the identifiability effect in these contexts may be related to the underlying relationship between the groups. While the relationships between the groups in the earlier study [30] were neutral, in the latter study the groups were in conflict. Intergroup conflict was pertinent both in the political setting and in the sports teams’ supporters—suggesting that generosity toward an individual member of one’s own in-group or out-group may be affected by the relationship between the two groups.

Earlier research [31; 32; 33] found that conflict situations lead both to assimilation within category boundaries and to contrast between categories—such that the members of the in-group are seen as more similar to the self than the members of the out-group. At the same time, encountering an individual out-group member may heighten their perceived uniqueness, thereby enabling them to be differentiated from other out-group members. In those situations, generosity toward the out-group individual would be greater if he or she is identified.

Other research suggests that in situations where group membership is salient—as when the groups are engaged in overt conflict—the individual tends to perceive both in-group and out-group members in a depersonalized manner by shifting from a personal to a social identity. Consequently, the in-group is viewed as coherent and homogeneous [34]. Under these circumstances, an unidentified, “generic” in-group member would be perceived as exemplifying the in-group more than an identified member, who necessarily possesses unique features, as well as the shared ones. This process may account for the greater generosity toward an unidentified (generic) in-group member relative to an identified one. By the same token, an unidentified, “generic” out-group member is likely to be perceived as exemplifying the opposing out-group more than an individual and identified out-group member—which may account for the weaker generosity toward an unidentified out-group member, relative to the identified one. Inter-group conflict often affects the perceived homogeneity and cohesiveness of the groups involved. In a recent study, Badea, Brauer, and Rubin [35] showed that group homogeneity is associated with group cohesiveness, and in intergroup contest situations winning groups are perceived as more homogenous and cohesive than losing groups. Returning to the comparison of the two basic social structures we employed in our recent studies of the identifiable victim effect, it may be argued that nationality-based groups—such as Israeli, Indian, or Argentinian—are perceived as less homogeneous and less cohesive than politically affiliated groups such as “doves” and “hawks.” It is possible, therefore, that the reversal of the identified victim effect depends on the perception of homogeneity and cohesiveness of the groups, rather than on the inter-group conflict in itself.

The present study aims to investigate the role of identifiability in a social context involving two non-rival groups. Unlike our previous research, in this case we examined novel groups, that were generated in the lab based on the *minimal group paradigm* [36]. This methodology creates arbitrary groups with no real meaning or history, based on the minimum conditions necessary for the creation of a sense of group membership. Thus, the grouping criterion is unrelated to nationality, political affiliation, or any other socially relevant attributes.

Our first goal was to test whether the interaction that was evident between identifiability and group affiliation in the case of nationality-based groups was replicated in a novel-group context in which the members of the novel group (based on the minimal group paradigm) bore no obvious relationship to one another. The second goal was to examine the role of group cohesiveness in explaining the reversal of the identifiability effect found in the case of rival groups, by examining whether minimal groups with greater cohesiveness but with no inter-group conflict also exhibited such a reversal.

Research suggests that group cohesion is associated with a sense of “we-ness”, or connectedness to a group as a whole [37; 38]. Intra-group connectedness was shown to increase cooperation between group members. Gaertner and Schopler [39] found that intra-group interactions resulted in more resources being allocated to in-group members. Similarly, strengthening group identity by emphasizing a common destiny led to greater cooperation between group members [40; 41; 42; 43]. These studies, however, did not examine the effect of interconnectedness on generosity toward identifiable versus unidentifiable members of other groups, so they offer no clues as to the role of identifiability in that context.

Concerns about how one’s altruistic decision is construed may be an important determinant of such a choice. People evaluate the kindness of an act not only by its consequences but also by its underlying intention [44; 45]. Offering help to an unidentified member of one’s in-group is more likely to be construed as supporting group members as a whole than when the prospective recipient is a specific identified member. The motivation to express one’s support for the group may be especially pertinent when group identity is salient, as in a highly interconnected group. Conversely, helping a specific individual who is not an in-group member is more easily interpreted as helping that particular person than helping out-group members.

In summary, we expect identifiability of the recipient to affect giving by an unconstrained allocator differently, depending on group-belonging and on the nature of the group. In particular, we predicted that in non-cohesive groups allocators would be more generous toward identifiable recipients who are members of their in-group, but not of their out-group. Conversely, in the case of groups with high cohesiveness and interconnectedness, we predicted that allocators would show more generosity toward unidentifiable in-group members relative to identifiable in-group members—while identifiability would have the opposite influence in relation to out-group members.

We examined these hypotheses in three experiments. In all of them we used the dictator game—a tool that has been extensively used in studies of altruistic, other-oriented behavior. In the standard version of this game, participants are randomly paired: in each pair, one (again randomly determined) receives an endowment and decides on how to allocate some (or all, or none) of the endowment to the other. Anonymity is preserved throughout the experiment, so that there is no personal contact between the allocator and the recipient, and no opportunity for reciprocation.

Contrary to the strict rational prediction, dictators do not maximize their own monetary payoff by keeping all the endowment for themselves. Instead, most people tend to give some of the endowment—about 20–30% on average—to their partner. In the present study we examined the effects of the recipients’ identifiability, group-affiliation, and group cohesiveness, on the respective dictators’ allocations.

## Experiment 1

In this experiment, the dictator game was played individually, using experimentally-generated groups. In each experimental session, we established two groups, based on Tajfel’s minimal group paradigm [36]. Participants first performed an “artistic preference” task, in which they were presented with three pairs of pictures, and asked to indicate, in each case, which of the two pictures they liked better. In each session, participants were then split into two groups of equal size (“Yellow” and “Blue”)—supposedly based on their response in the artistic preference task. In reality, they were assigned to the two groups at random.

Next, they each played a dictator game with an anonymous partner, who belonged either to the same group as themselves (the *In-group* condition) or to the other group (*Out-group condition*). In addition, the recipient was either identifiable by an experimentally-assigned number, or unidentifiable (their number to be determined at the end of the experiment). Thus, the experimental design included a 2 (In-group versus Out-group) × 2 (identifiable versus unidentifiable) between-subject design. To the extent that our earlier findings involving (neutral) nationality-based social groupings might be generalized to the present situation, we expected identifiability to increase allocation to an in-group recipient but not to an out-group one.

### Method

One hundred and seven students took part in the study (51 men, 56 women; mean age 23.4)—each receiving 15 shekels as a show-up fee (in addition to the amount earned during the dictator game). Sample size was determined before any data analysis. The experiment was conducted in 7 sessions, each involving 13–16 participants. Participants first completed an Artistic Preference questionnaire, then randomly assigned to one of the two groups (“Blue” or “Yellow”) by receiving a personal note with their group’s affiliation—supposedly based on their answers to the questionnaire, but in reality, determined at random. To verify that the grouping manipulation affected participants’ perception as intended, each participant was asked two questions: 1) *If you were to receive a gift from a participant in this experiment*, *are you more likely to like it if it were chosen by a participant from the Yellow group*, *or from the Blue group*? and 2) *If the groups were given a task to make a wall graffiti*, *which of the two groups would produce a more original picture*?

Next, participants were introduced to the dictator game, in which half of them would receive 11 shekels (in one shekel coins), and had to decide whether to share the money with their allotted partner. A lottery was held to determine who would receive the money, by having each participant draw one envelope from a pile of sealed envelopes containing the questionnaires. Each questionnaire stated their role (allocator or recipient) and information about their partner’s group affiliation and identifiability (in accordance with the experiment’s condition). For example, in the Identifiable condition, the questionnaire for each allocator read: *You have been randomly chosen to play Role A* (the allocator). *You receive 11 shekels*. *Your partner has been randomly chosen from the Yellow group*. *His/her number is 7* (or *His/her number will be randomly determined later on*, in the Unidentifiable condition). After reading the instructions, each allocator decided how much money, if any, they would share with their partner. They then placed that amount in an envelope, along with their completed questionnaire, in which they were asked to estimate how much money their partner would have left them, had their roles been switched. Non-allocators were also given a questionnaire to complete, in which they were asked how much money they expected to receive from their partner, and how much they would have given their partner had the roles been switched. Since non-allocators also placed their questionnaire in the envelope neither the role played by the participants nor their decision (in case of the allocators) were publicly apparent. The envelopes with the money shared by the allocators were given to their respective recipients at the end of the experimental session.

This experiment, as well as Experiments 2 and 3 were approved by the Hebrew University, School of Education Ethics committee. All participants were adults and have signed a written consent before their participation in the studies. We report all measures, manipulations and exclusions.

### Results

We first examined the responses to the manipulation check questions. Seventy-four percent of the participants expected to like a gift from an in-group member more than a gift from an out-group one (Chi-square = 24.51, *p* < .001). Similarly, 66% of the participants thought that their group was likely to produce the more original wall graffiti (Chi-square = 10.57, *p* = .001). Thus, overall, participants appear to have recognized the distinctive qualities of the two groups and to conform to their group membership. Six participants whose answers to both questions revealed a preference for the out-group were eliminated from further analyses. We note that the elimination of these six participants was unrelated to anticipated or expressed generosity, as their reservations about their fellow members’ taste in gifts or artistic ability likely indicated a lack of identification with their allotted group.

**Fig 1:**
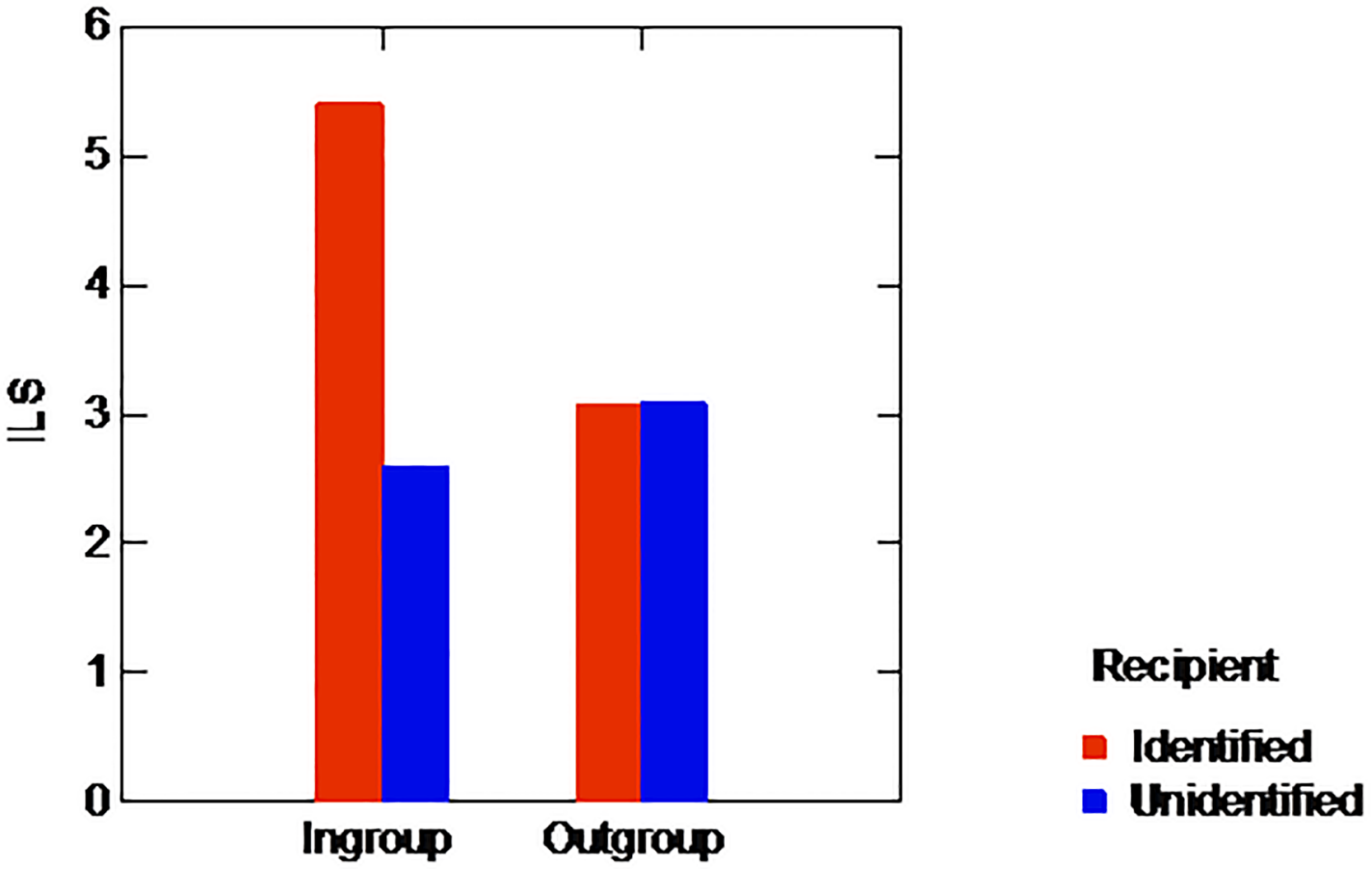
Mean allocation by recipient’s group-affiliation and identifiability, Experiment 1.

We start by examining the allocators’ behavior, since our primary focus was on the actual amounts that they gave their respective partners. Nine of the 50 allocators gave their partner nothing at all. This proportion of zero-allocators did not significantly vary by condition (16% and 20% for the In-group and Out-group conditions, respectively; Chi-square = .713, p = .50). The mean amounts (using ILS, the conventional acronym for shekel) given by the allocators (including the empty envelopes) in the various conditions are depicted in Fig 1. An analysis of variance (ANOVA) of the amount transferred by group affiliation and identifiability yielded a significant main effect of identifiability (*F*(1,46) = 3.863, *p* = .05, *η*_*p*_^*2*^ = .077), qualified by a significant interaction with group affiliation (*F*(1,46) = 3.972, *p* = .05, *η*_*p*_^*2*^ = .079). As clearly evident from the figure, the effect of identifiability was limited to in-group recipients: identifiable in-group recipients received larger allocations than their unidentifiable counterparts (5.400 versus 2.600, respectively; *t*(23) = 2.568, *p* < .05), while identifiability had no apparent effect on the allocation to out-group recipients (3.071 versus 3.091 for identifiable and unidentifiable recipients respectively, t(23) = .022, p = .98).

Since the sample of allocators is small, we carried out a nonparametric bootstrap test of the interaction in addition to this analysis. A total of 100,000 bootstrap samples were taken from the original data after correcting for the estimated interaction. Only in 6% of these the estimated interaction was higher than in the original data. This result supports the parametric analysis finding of (marginally) significant interaction. Notably, the *q-q* plot of the 100,000 estimated interactions shows a very good fit for the normal distribution, thereby justifying the use of the standard parametric test (for which we assume the normality of the estimate, not of the data).

Including in the analysis the actual amounts given by the allocators and the hypothetical allocation by the non-allocators (in reply to the question as to how much they would have given their partner had the roles been reversed) did not change the findings significantly. The proportion of zero allocations still did not significantly vary by condition (14.3% and 15.4% for the In-group and Out-group respectively; Chi-square = .877, p = .55). Next, we ran an ANOVA of the allocations (either actual or hypothetical) by group affiliation, identifiability, and player role (allocator versus recipient). We first noted that player role had no effect, and did not interact with the other factors, enabling us to draw conclusions based on both real and hypothetical responses. Next, the ANOVA yielded a significant main effect of identifiability (4.265 versus 3.176 for identifiable and unidentifiable recipients, respectively; *F*(1,92) = 5.681, *p* < .05, *η*_*p*_^*2*^ = .058) and a significant interaction (*F*(1,92) = 5.446, *p* < .05, *η*_*p*_^*2*^ = .056)—whereby identifiable in-group recipients received larger allocations than unidentified ones (5.157 versus 2.827, t(46) = 3.152, p < .005), while there was virtually no difference in allocations to identifiable versus unidentifiable out-group recipients (3.700 versus 3.636, t(50) = .099, p = .921). No other effects or interactions approached statistical significance. The interaction of identifiability and group affiliation remained nearly significant when all participants were included in the analysis (*F*(1,98) = 2.841, *p* = .09, *η*^*2*^ = .028).

We next examined the participants’ expectations as recipients. Two versions of the expectations question were used: recipients were asked *How much money will the allocator leave you*? and allocators were asked *How much money would you expect the recipient to leave you*, *if he/she were the allocator*? (Note that in both cases the question pertained to the respondent’s *expectations*, rather than to their respective partner’s actual behavior.) Not surprisingly, perhaps, participants tended to consider themselves more generous than their partners: on average, the within-subject comparison of allocation and receipt expectations showed the former to be higher than the latter (3.700 versus 3.030, for expectations of allocation versus receipt; *t*(99) = 3.169, *p* < .005).

A more interesting question for the present study was whether expectations of receiving allocations exhibited the same interaction pattern as the allocation decisions. An ANOVA of participants’ expectations by identifiability, group-affiliation, and player role yielded a significant interaction of group affiliation and identifiability (*F*(1,92) = 9.858, *p* < .005, *η*_*p*_^*2*^ = .097)—whereby participants in the identifiable In-group condition expected to receive larger amounts than those in the unidentifiable In-group condition (4.053 versus 2.310, respectively; *t*(46) = 2.742, *p* < .05). Expectations of identifiable and unidentifiable participants in the Out-group condition did not differ significantly (2.600 versus 3.682 for identifiable and unidentifiable participants, respectively; t(50) = 1.671, p = .101). The similar patterns in expectations and actual outcomes suggest that there is a consistent differential impact of identifiability on respondents when recipients are fellow in-group members rather than out-group members.

The results of the first study replicated the pattern found in our earlier research on the effect of identifiability in inter-group contexts, when groups were based on nationalities, and relations between the nations in question were neutral (i.e., they were not in conflict). In that context, identifiability increased the sharing behavior only when the recipient belonged to the in-group. In both the nationality-based groups in the former study and in the groups based on the minimal group paradigm in the current study, group cohesiveness was low, since no real familiarity existed between in-group members. In the next experiment we used the same minimal group paradigm, while introducing a preliminary stage designed to enhance group cohesiveness. In line with our hypothesis, we expected the enhanced group cohesiveness to yield a reversal of the identifiability effect—namely, greater generosity toward identifiable out-group members, but not toward identifiable in-group members.

## Experiment 2

To investigate the role of the recipient’s identifiability in cohesive groups, we conducted an experiment that replicated the design of Experiment 1, with the same minimal groups, but this time with enhanced group cohesiveness. To this end, we introduced a collaborative task with a shared group goal prior to playing the dictator game. Such activity is known to increase group cohesion [46]. To ensure that effect, we first conducted a pilot study to see if the task we used did indeed increase participants’ perceptions of the group’s cohesiveness.

### Pilot study

#### Method

Thirty-two undergraduate students (51% female; mean age 24.3) took part in the pilot study that was conducted over two sessions, in exchange for 15 shekels. Participants registered for the study via the lab’s web page. During their registration, they were randomly assigned to one of two order-conditions (*Before* and *After*): in one session participants completed a questionnaire of subjective perceptions of group cohesiveness before being engaging in the collaborative task; while in the other session they completed the same questionnaire after participating in the task. As in Experiment 1, participants in both order-conditions first completed the Artistic Preference questionnaire, after which they were randomly assigned to one of the two groups (Blue or Yellow), although it was supposedly based on their answers to the questionnaire. Participants were then seated—each group in a different corner of the room—and engaged in a group activity with their fellow group members. The task was based on the animated book *Where's Waldo*?, in which participants were instructed (as a group) to find as many specified characters as possible on a large poster, in a fixed short time. Participants then completed a Group Cohesion questionnaire either before or after the group’s activity (in accordance with the session’s order-condition). This comprised six items from two common group-cohesion scales that are relevant to the context of the current groups. Two items were taken from the Work Team Cohesion Scale [47]. *This team is united in trying to reach its goals* and *I'm happy with this team’s level of commitment to its tasks*). Two items were taken from the Perceived Cohesion Scale [37]: *The members of this group feel a sense of participation* and *I feel accepted by the group*. Finally, two items examined perceived similarity and connectedness between group members [48] by asking participants to rate the degree to which they would use the term “*we*” to describe themselves and their fellow group members, and the degree of perceived similarity between the group’s members. All six questions were rated on a 7-point scale ranging from *1* –*Not at all* to *7 –Very much*. Since Cronbach’s alpha of the six items was 0.897, we computed for each participant the mean cohesiveness perception.

#### Results

An independent t-test revealed a highly significant difference in subjective perceptions of group cohesiveness between groups that had evaluated their cohesiveness before (M = 3.42) and after (M = 5.11) engaging in the collaborative task (*t*(30) = 4.71, *p* < .001). The results of the pilot test support the claim that the collaborative task increases the sense of group cohesiveness. We therefore used this task to increase the sense of group cohesiveness in Study 2.

### Main experiment

#### Method

A total of 80 students participated in this study (40 men, 40 women; mean age 24.8). The experiment was conducted over 5 sessions, with 14–18 participants in each. As in Experiment 1, participants first completed the Artistic Preference questionnaire, then were randomly assigned to one of the two groups (Blue or Yellow)—supposedly based on their answers to the questionnaire, but in reality determined at random. The same two questions were used as in Experiment 1, to verify that the grouping manipulation had the intended effect on the participants’ perception.

After answering the two questions, participants were seated, each group in a different corner of the room. To increase a sense of group cohesiveness, participants then engaged in the group activity described in the pilot study. They were then introduced to the dictator game, and the session proceeded exactly as in Experiment 1.

#### Results

We first examined the response to the manipulation check questions. Eighty percent of the participants expected to like a gift from an in-group member more than a gift from an out-group participant (Chi-square = 27.86, *p* < .001). Similarly, 70% of the participants thought that their group was likely to produce the more original wall-graffiti (Chi-square = 12.80, *p* < .001). Overall, therefore, participants appeared to recognize the distinctive qualities of the two groups and to conform to their group membership. Four participants whose answers to both questions showed a preference for the out-group were removed from further analyses. (As in Experiment 1, here too this was unrelated to their anticipated or explicit generosity, but rather to their reservations about their fellow group members’ taste in gifts or artistic abilities.)

**Fig 2:**
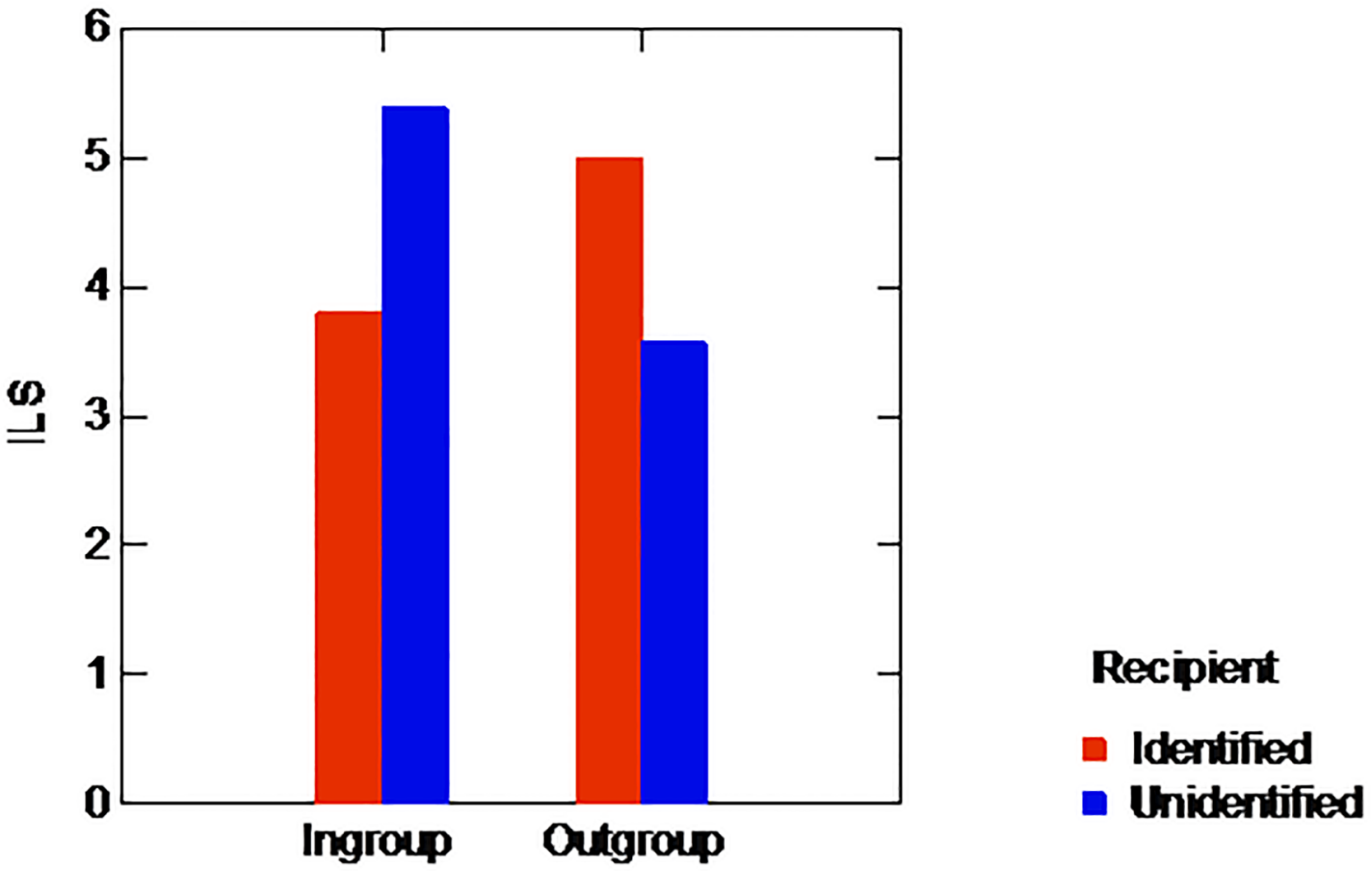
Mean allocation by recipient's group affiliation and identifiability, Experiment 2.

As in the previous experiment, we began our analysis by examining the amount of money actually left by the allocators for their respective recipients. Four of the 38 allocators had left their partner no money whatsoever. The proportion of such zero allocators did not significantly vary by condition (8.7% and 13.3% for the In-group and Out-group respectively; chi-square = .649, p = .52). The mean amounts given by the allocators (including zero allocations) in the various conditions are displayed in Fig 2. An ANOVA of the allocations by group affiliation and identifiability of the recipient yielded no significant main effect either of group affiliation (F(1,33) = .257, p = .616) or of identifiability (F(1) = 33 = .017, p = .898). However, as predicted, the analysis did reveal a significant interaction of identifiability and group affiliation (*F*(1,33) = 6.204, *p* < .05, *η*_*p*_^*2*^ = .158). As the figure clearly shows, the impact of identifiability on the allocations varied with the recipient’s group affiliation: identifiable in-group recipients received lower allocations than unidentified ones (3.800 versus 5.385 respectively, (*t*(21) = 2.527, *p* < .05), while the mean allocation to identifiable out-group recipients tended to be higher than the mean allocations to unidentifiable out-group recipients—albeit to a non-significant degree (5.000 versus 3.571, respectively, t(12) = 1.219, p = .246). As in Experiment 1, here too, in addition to this analysis, a nonparametric, bootstrap test of the interaction was carried out—in the same manner as in Experiment 1. We found that only in 0.028 of the data sets generated in the bootstrap procedure was the interaction larger than the interaction computed in the actual data set. Here too, the q-q plot of the 100,000 estimated interactions showed a very good fit for the normal distribution—thereby justifying the use of the standard normal test.

As in Experiment 1, a combined analysis including both the actual allocations by the allocators and the hypothetical allocations of the non-allocators yielded similar results. An ANOVA of the allocations (either actual or hypothetical) by group affiliation, identifiability, and player role (allocator versus recipient) yielded a marginally significant 2-way interaction of identifiability and group affiliation (*F*(1,67) = 3.668, *p* = .06, *η*_*p*_^*2*^ = .052)—whereby identifiable in-group recipients received marginally lower allocations than unidentified ones (4.00 versus 5.33, *t*(41) = 1.761, *p* = .08). The mean allocation for identifiable out-group recipients was higher than the corresponding mean for unidentifiable out-group recipients—however, this difference was not statistically significant (4.875 versus 4.062, t(30) = 1.019, p = .317). No other effects or interactions approach a significant level. The interaction of identifiability and group affiliation remained nearly significant when all participants were included in the analysis (*F*(1,71) = 3.231, *p* = .07, *η*^*2*^ = .044).

We next examined the participants’ expectations as recipients. Once again, we found that participants thought themselves more generous than their partners: on average, the within-subject comparison of allocation and reception expectations showed the former to be higher than the latter (4.59 versus 3.78, for allocation versus reception expectations; *t*(73) = 3.479, *p* < .005). Otherwise, none of the manipulated factors had a significant impact on expectations.

### Direct comparison of Experiment 1 and Experiment 2

Experiment 1 and Experiment 2 were run on different occasions, so there was no random assignment of participants to experiment (#1 or #2). However, identical procedures were employed in each case, with exception of the intended difference of cohesiveness manipulation—the intra-group collaborative task. Furthermore, participants in the studies came from similar population samples. While taking into account concerns about non-random assignment, we believe analysis of the combined data from the two experiments offers further support for the effect of group cohesiveness and its interaction with target identifiability. To this end, we integrated the data from the two experiments, for cohesiveness (the conditions of Experiment 1 being coded as *low-cohesiveness* and those of Experiment 2 as *high-cohesiveness*).

An ANOVA of allocators’ allocations by identifiability, group affiliation, and cohesiveness yielded a marginally significant effect of cohesiveness—suggesting that overall, increasing cohesiveness increased allocations across all conditions (4.540 versus 3.400 for high versus low cohesiveness, respectively; *F*(1,79) = 3.354, *p* = .07, *η*_*p*_^*2*^ = .041). Importantly, the analysis revealed a highly significant three-way interaction of cohesiveness, identifiability and group affiliation (*F*(1,79) = 8.836, *p* < .005, *η*_*p*_^*2*^ = .101). No other effects reached statistical significance.

Repeating the analysis (as we did in both experiments)—including both the actual allocations by the allocators and the hypothetical allocations by the non-allocators—yielded similar results. An ANOVA of the allocations (either actual or hypothetical) by group affiliation, identifiability, player role (allocator versus recipient), and cohesiveness once again yielded a marginally significant main effect of cohesiveness on allocation amounts (*F*(1,159) = 3.736, *p* = .05, *η*_*p*_^*2*^ = .023) and a highly significant three-way interaction of identifiability, group affiliation, and cohesiveness (*F*(1,159) = 8.869, *p* < .005, *η*_*p*_^*2*^ = .053). No other effects yielded statistically significant results. Interestingly, the experimental manipulations affected behavior toward an in-group member more than toward an out-group one. Following this analysis, by analyzing the In-group and Out-group conditions separately, we found no significant effect or interaction (for cohesiveness or for identifiability) in the Out-group condition, while in the In-group condition the interaction of identifiability with cohesiveness was highly significant (*F*(1,83) = 11.156, *p* = .001, *η*_*p*_^*2*^ = .118): in non-cohesive groups, identifiable in-group members received higher amounts than unidentifiable ones (5.158 versus 2.828, respectively, t(46) = 3.152, p < .005), while in cohesive groups, identifiable members received (marginally significant) lower amounts than unidentifiable ones (4.000 versus 5.333, respectively, t(41) = 1.761, p = .08). These results suggest that the effect of identifiability on generosity toward recipients—particularly in-group ones—varies according to the group’s cohesiveness.

However, since group cohesiveness was not manipulated or measured within the same study, this conclusion must be interpreted with caution. In the next experiment, we further examined the effect of cohesiveness on in-group recipients.

## Experiment 3

This experiment was designed to further examine the role of perceived cohesiveness of one’s group and its effect on participants’ generosity toward identifiable and unidentifiable in-group recipients. For this study, we used a natural setting of existing groups, and measured their subjective perception of cohesiveness. This allowed us to directly examine the effect of perceived group cohesion on the incidence of sharing with a partner who is either identified or unidentified.

### Method

Ninety seven undergraduate students (90% females; mean age = 24.14, SD = 4.27^i^) took part in the study for course credit. The study took place at the end of a Research Methods exercise session, involving small groups of 18 to 25 students, who meet once a week throughout the semester.

Participants were told that the experiment consisted of two (supposedly) unrelated tasks. First, they completed the Group Cohesiveness questionnaire (as described in Study 2) with respect to their Research Methods exercise group. As the Cronbach’s alpha of the six items was 0.83, we computed the mean cohesiveness perception for each participant.

Next, each participant was paired up with another student from their exercise section (an in-group partner), and introduced to the dictator game which they were to play with their respective partners. In the game, half of them received 11 shekels and asked to decide whether or not to share that money with their partners. (As in the previous studies, a lottery was held to determine who received the money, by having each of participant draw an envelope from a pile of sealed envelopes containing the questionnaires.) Participants were then randomly assigned to share with either an identifiable or an unidentifiable recipient (using the same manipulation as in the previous studies). Participants in the recipient role were asked how much they would have allocated to their partner had they been assigned the allocator role. The rest of the experiment proceeded precisely as in the previous studies.

### Results

We began our analysis by examining the amount of money actually left by their allocators for their respective partners. Five of the 50 allocators returned empty envelopes, having left their partner no money whatsoever. The proportion of such zero allocators did not significantly vary by condition (8.7% and 11.1% for the In-group and Out-group, respectively; Chi-square = .081, p = .77). To examine the role of Identifiability of partner (identifiable or unidentifiable) and of the Cohesiveness rating in predicting the amount of money shared with one’s partner, we conducted a regression analysis with identifiability, cohesiveness, and the interaction between those variables as predictors. The regression analysis yielded no significant results. In particular, although the correlation between cohesiveness and allocations was more negative in the Identifiable than in the Unidentifiable condition (r = -.329 and -.029, respectively), the interaction of identifiability and cohesiveness was not statistically significant (F(1,46) = .978, p = .328).

As in the previous experiments, we combined the allocators’ actual allocations with the recipients’ hypothetical ones, to form a single measure of allocation to one’s partner. We repeated the above analysis, with Player role (allocator or recipient) added as a third independent variable, in addition to Identifiability of partner and Cohesiveness rating. The predictors included all three main effects (Identifiability, Player and Cohesivenes*s*); all two-way interactions; and the three-way interaction between those variables. The results of this analysis are presented in Table 1. We note first that the player’s role did not significantly affect the allocation, nor did it interact with identifiability or cohesiveness (p > .15 for each). The analysis also revealed a significant main effect of identifiability—whereby identifiable recipients were allocated higher amounts than unidentifiable ones (F(1,89) = 4.561, p < .05). The main effect of judged cohesiveness was not significant (p > .6).

**Table 1:** General linear model for predicting allocation N- = 97 multiple R: 0.298 squared multiple R: 0.089.

	B	Sum-of-Squares	df	Mean-Square	F-ratio	P
Cohesiveness	0.115	1.586	1	1.586	0.255	0.615
Identifiability (1 = ident.; 0 = unident.)	2.186	28.380	1	28.380	4.561	0.035
Player (1 = dictator; 0 = recipient)	1.337	10.605	1	10.605	1.704	0.195
Identifiability*Cohesiveness	-0.463	25.897	1	25.897	4.162	0.044
Player*Cohesiveness	-0.328	12.948	1	12.948	2.081	0.153
Player*Identifiability	-0.641	2.436	1	2.436	0.391	0.533
Player*Cohesiveness*Identifiability	0.098	1.151	1	1.151	0.185	0.668
Error		553.777	89	6.222		

**Fig 3:**
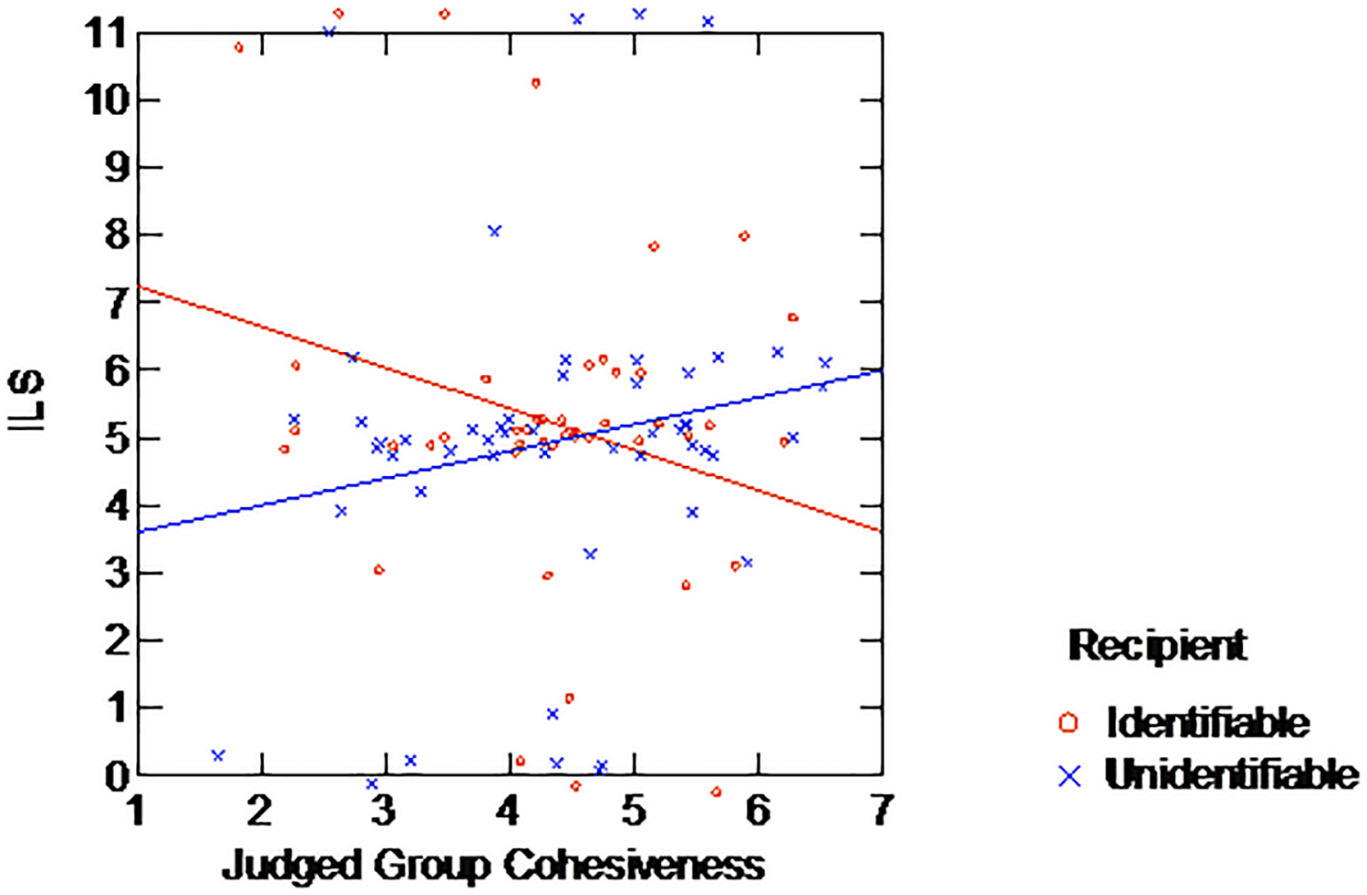
Mean allocation by recipient's identifiability and perceived group's cohesiveness, Experiment 3.

Most importantly, the interaction of interest between Identifiability and Cohesiveness was significant (F(1,89) = 4.162, p = .044). This interaction is plotted in Fig 3. As it clearly shows, the effect of cohesiveness on allocation differs between identifiable and unidentifiable recipients: while allocation to an identifiable recipient appears to decrease with perceived group cohesiveness, allocation to an unidentifiable one increases, although not significantly so (r = -.258, p = .07 and r = .189, p = .17, respectively).

In summary, the results of the present experiment are consistent with the hypothesis that in the context of an in-group, the more cohesive the group, the lower the generosity toward an identifiable group member, compared with an unidentifiable one.

## Discussion

The present study demonstrates that an identifiable victim does not always trigger a greater willingness to help than an unidentifiable one. Our findings—both current and previous—outline a complex picture regarding the role of a recipient’s identifiability in group settings, whereby the nature of inter- and intra-group relations determines the effect of identifiability on the generosity of the donor.

While in our earlier research we examined national groups [30] and groups in conflict [6], in the current study we used the pure manipulation of the minimal group paradigm to examine the role of identifiability in that context. In this context, generosity was measured by the amount given by allocators to their respective recipients in a dictator game. The effect of identifiability was tested in dictator games. Allocators were paired with recipients who were either fellow in-group or out-group members, and either identifiable or unidentifiable. In Experiment 1—which did not include a cohesiveness enhancing activity (such as a collaborative task) prior to the game—identifiability increased allocation to an in-group recipient, but not to an out-group one. By contrast, in Experiment 2, where the dictator game was played after a joint activity designed to enhance group cohesiveness, the identifiability effect was reversed: allocators were more generous toward their unidentifiable partners than toward identifiable in-group members—and the opposite was true for out-group recipients. In Experiment 3, the role of perceived group cohesiveness was directly examined by having students play the dictator game with partners from their own exercise section, and their perception of the section as a cohesive group was measured. Identifiability resulted in increased sharing between participants who saw their section as low in cohesiveness, but not among those who viewed it as a highly cohesive group.

The findings of these studies shed light on the earlier conflicting results concerning the role of identifiability in inter-group contexts. In particular, they suggest that increased generosity toward identified compatriots that does not extend to identified victims of other nations [30] is an instance of a more general social categorization effect, whereby in non-cohesive groups identifiability increases allocation to in-group recipients, but not to out-group ones. Conversely, they also indicate that diminished generosity toward an identifiable in-group recipient compared with an unidentified one—as evident in groups embroiled in inter-group conflict [6]—can be accounted for by the cohesiveness of the groups. External competition or conflict increases internal cohesion, because when group members perceive there to be an active rivalry with another group, they tend to view their own group as a means of overcoming an external threat. This process increases perceptions of in-group cohesiveness [49].

Increased group cohesiveness may be achieved through interactions between in-group members and working together toward a common goal, or by experiencing external competition. In either case, boosted cohesiveness is expected to increase a sense of connectedness, making the group as a whole more meaningful. Due to the tendency to highlight prototypical, depersonalized characteristics of the group, group cohesiveness is associated more with attraction to the group as a whole than with interpersonal attraction between individuals within the group [50].

One construct related to perceived group cohesiveness is that of *entitativity*—or extent to which a group can be seen as “an entity.” Recent findings [51] suggest that in-groups are perceived to be relatively more entitative than out-groups (although their members are not judged more similar to each other than members of the out-group). By contrast, out-group members were perceived to be more alike. Assuming that cohesive groups are regarded as more entitative than non-cohesive ones, Crump et al.’s finding suggests that cohesiveness may strengthen the idea of a group at an abstract, perhaps prototypical level—without increasing the degree to which any specific group member is perceived to exemplify the group membership. Furthermore, in cohesive groups, more than in non-cohesive ones, entitativity may cause shared features to loom large in perceived similarity [52]. Accordingly, any specific in-group individual—who necessarily possesses unique features in addition to the shared ones (i.e., an identifiable specific in-group member)—may be perceived as being less similar to the allocator than a generic, unidentifiable one. These findings regarding entitativity suggest—as indeed we have found—that in cohesive groups allocations to fellow in-group members are greater at the group level (i.e., to unidentified, depersonalized recipients) than at the individual level (i.e., to an identified recipient)—while allocations to out-group members at the group level are smaller than at the individual level.

Besides its theoretical contribution to our understanding of the role of group cohesiveness as a key factor in altruistic behavior toward identifiable and unidentifiable victims, our research suggests practical implications for increasing the frequency and scope of pro-social behavior. Specifically, our findings offer insights regarding possible ways to increase caring for out-group victims, depending on the perceived cohesiveness of the group in question (for example, by focusing on a specific out-group member in donation requests, when the donors’ perceived group cohesiveness is strong). However, while in some instances improving attitudes toward a specific out-group member may improve attitudes toward the out-group as a whole [53], people may behave in a less biased way toward a specific out-group member (i.e., show less discrimination) to justify their negative treatment of other out-group members [22]. Future research should examine how the increased willingness to share with a specific identified out-group member affects the donor’s attitude to the out-group as a whole.

## Supporting information

S1 Data PON-Ex1.sav. Data of Ex1.
(SAV)

S2 Data PON-Ex2.sav. Data of Ex2.
(SAV)

S3 Data PON-Ex3.sav. Data of Ex3.
(SAV)
